# Complete Genome Sequence of Feline Calicivirus Strain HRB-SS from a Cat in Heilongjiang Province, Northeastern China

**DOI:** 10.1128/genomeA.00698-14

**Published:** 2014-09-04

**Authors:** Chunguo Liu, Yongxiang Liu, Dafei Liu, Dongchun Guo, Ming Liu, Yijing Li, Liandong Qu

**Affiliations:** a State Key Laboratory of Veterinary Biotechnology, Harbin Veterinary Research Institute, Chinese Academy of Agricultural Sciences, Harbin, Heilongjiang, China; b College of Veterinary Medicine, Northeast Agricultural University, Harbin, Heilongjiang, China

## Abstract

Here, we report the complete genome sequence of feline calicivirus (FCV) strain HRB-SS isolated in 2014 from a cat in Heilongjiang Province of northeastern China. The virus genome is 7,705 bases, excluding the poly(A) tail. This analysis will help to understand the genetic characteristics and evolution of FCV in China.

## GENOME ANNOUNCEMENT

Feline calicivirus (FCV) is one of members of the genus *Vesivirus*, in the family *Caliciviridae*, and is also an important infectious pathogen of cats that can cause a range of severe clinical symptoms (1). The FCV genome contains a positive-sense single-stranded RNA and is apt to produce genetic and antigenic mutations, which may be responsible for the virulence differences and the failure of vaccines (2). Therefore, based on previous studies, the whole-genome analysis is particularly important.

In the present study, the FCV HRB-SS strain was isolated in 2014 from a cat with eye disease in Heilongjiang Province, northeastern China. The diseased cat developed the symptoms of severe keratitis, photophobia, and clear nasal discharge flow; however, there were no other clinical symptoms, which was somewhat different from the clinical symptoms previously reported with FCV (1). Gerriets et al. (3) reported that FCV was primarily an ocular pathogen causing ocular surface disorders in cats. In this study, we analyzed the full-length genome of FCV HRB-SS strain to discover the genetic differences. Swabs of the nose and eye were immersed in phosphate-buffered saline and vibrated vigorously, then centrifuged at 12,000 × *g* for 10 min at 4°C, and the supernatant was filtrated by a 0.22-μm syringe filter. Following the filter liquor were inoculated with a Crandell-Rees feline kidney (CRFK) cell monolayer, and the viruses were harvested for RNA extraction. cDNA was synthesized using an oligo(dT) primer. The 2.5-kb, 2.9-kb, and 2.4-kb overlapping cDNA fragments covering the complete genome were amplified with KOD-Plus-Neo DNA polymerase (Toyobo), cloned into the pMD18-T vector, and the positive clone were sequenced by vector primers and FCV-specific primers by Invitrogen, Shanghai branch.

The genome of FCV HRB-SS consists of 7,705 nucleotides (nt), excluding the poly(A) tail. The bioinformatics analysis showed that the FCV HRB-SS strain contains two large open reading frames (ORFs), ORF1 (nt 20 to 5311) and ORF2 (nt 5312 to 7318), as well as one small ORF, ORF3 (nt 7315 to 7638). The 5′- and 3′-untranslated regions are 19 nt and 67 nt long, respectively. The nucleotide sequence of FCV HRB-SS showed the highest identity (80.3%) with 1874 strain (accession no. JX519214), while the identity was only 76.6% with the GD strain (accession no. GU214989) isolated from China, which was the lowest among all the complete genomes of FCV deposited in GenBank. Phylogenetic analysis showed that FCV HRB-SS is closely related to FCV/DD/2006/GE and UTCVM-H2, which colocated in the same cluster with FCV U.S. strains Urbana (accession no. NC001481), 1874 (accession no. JX519214), and 5789 (accession no. JX519210). Compared to the vaccine strain F9 (accession no. M86379), FCV HRB-SS has 78.7% nucleotide homology and is located on a different cluster; furthermore, the genome of FCV HRB-SS was inserted 24 nt into the 3′-end, which was similar to the South Korean isolates 12Q087-1 (accession no. KJ572400) and 12Q087-5 (accession no. KJ572401); however, the GD strain kept the same pattern as the F9 strain. The function remains unknown, and further studies should be carried out.

### Nucleotide sequence accession number.

The genome sequence of the FCV HRB-SS strain has been deposited in GenBank under the accession no. KM016908.
